# The Impact of Frailty and Surgical Risk on Health-Related Quality of Life After TAVI

**DOI:** 10.3390/jcdd11100333

**Published:** 2024-10-18

**Authors:** Kim E. H. M. van der Velden, Bart P. A. Spaetgens, Wolfgang F. F. A. Buhre, Bart Maesen, Dianne J. D. de Korte-de Boer, Sander M. J. van Kuijk, Arnoud W. J. van ‘t Hof, Jan U. Schreiber

**Affiliations:** 1 Department of Anesthesiology and Pain Medicine, Maastricht University Medical Center+ (MUMC+), 6229 HX Maastricht, The Netherlands; dianne.de.korte@mumc.nl (D.J.D.d.K.-d.B.); j.schreiber@mumc.nl (J.U.S.); 2 Cardiovascular Research Institute Maastricht (CARIM), Maastricht University, 6200 MD Maastricht, The Netherlands; arnoud.vant.hof@mumc.nl; 3 Department of Internal Medicine, Division of General Internal Medicine, Section Geriatric Medicine, Maastricht University Medical Center+ (MUMC+), 6229 HX Maastricht, The Netherlands; bartholomeus.spaetgens@mumc.nl; 4 Department of Anesthesiology, Division of Vital Functions, University Medical Center Utrecht (UMCU), 3584 CX Utrecht, The Netherlands; w.f.f.a.buhre@umcutrecht.nl; 5 Department of Cardiothoracic Surgery, Maastricht University Medical Center+ (MUMC+), 6229 HX Maastricht, The Netherlands; b.maesen@mumc.nl; 6 Department of Clinical Epidemiology and Medical Technology Assessment, Maastricht University Medical Center+ (MUMC+), 6229 HX Maastricht, The Netherlands; sander.van.kuijk@mumc.nl; 7 Department of Cardiology, Division of Interventional Cardiology, Maastricht University Medical Center+ (MUMC+), 6229 HX Maastricht, The Netherlands; 8 Department of Cardiology, Division of Interventional Cardiology, Zuyderland Medical Center, 6419 PC Heerlen, The Netherlands

**Keywords:** transcatheter aortic valve implantation, aortic stenosis, health-related quality of life, frailty

## Abstract

Symptomatic aortic stenosis and frailty reduce health-related quality of life (HrQoL). Transcatheter aortic valve implantation (TAVI) in patients at high to extreme risk has been proven to have a beneficial effect on HrQoL. Currently, TAVI is also considered in patients at intermediate risk. Our meta-analysis investigates whether benefits to HrQoL after TAVI is more pronounced in frail patients and patients at high to extreme vs. intermediate surgical risk. A systematic search of the literature was performed in November 2021 and updated in November 2023 in PUBMED, EMBASE, and the Cochrane Controlled Trials Register. Statistical analysis was performed according to the inverse variance method and the random effects model. A total of 951 studies were assessed, of which 19 studies were included. Meta-analysis showed a mean increase in the Kansas City Cardiomyopathy Questionnaire (KCCQ) score of 29.6 points (6.0, 33.1) in high to extreme risk patients versus 21.0 (20.9, 21.1) in intermediate risk patients (*p* < 0.00001) and 24.6 points (21.5, 27.8) in frail patients versus 26.8 (20.2, 33.4) in the general TAVI population (*p* = 0.55). However, qualitative analyses of non-randomized studies showed the opposite results. In conclusion, TAVI improves HrQoL more in high to extreme than intermediate risk patients. Frailty’s impact on HrQoL post-TAVI is inconclusive due to varying outcomes in RCTs vs. non-RCTs.

## 1. Introduction

Severe, symptomatic aortic stenosis (AS) is associated with an increased risk of mortality and a reduced health-related quality of life (HrQoL) compared to patients with asymptomatic AS or to the general older population [1,2]. Impaired HrQoL in patients with symptomatic AS is often a result of chest pain, dyspnea, syncope, limited exercise tolerance, and functional decline [1]. Treatment modalities for AS include surgical aortic valve replacement (SAVR), interventional procedures, such as transcatheter aortic valve implantation (TAVI) and balloon aortic valvuloplasty (BAV), and, finally, medical treatment for symptom relief. TAVI is considered a treatment alternative to SAVR for patients with severe symptomatic AS and high to extreme surgical risk, with similar results [3,4,5,6]. Despite significant reductions in 30-day and 1-year mortality rates after TAVI, over the last 10 years, rates of poor or reduced HrQoL remain high [7,8]. Therefore, it is important to understand which factors contribute to these adverse health outcomes in this subset of patients, e.g., those who die, do not improve, or even deteriorate in HrQoL after TAVI. While little is known about the factors driving excess mortality or deteriorated HrQoL, frailty has already proven to be associated with increased mortality and poor(er) HrQoL [9,10,11,12,13,14,15,16]. Studies on the impact of frailty are limited and show different results regarding its potential impact on HrQoL [12,13,14,15,16]. Some studies suggest that HrQoL tends to increase even more in frail patients, given their worse starting point, compared to non-frail patients, who report a significantly better HrQoL preoperatively, resulting in a less pronounced increase in HrQoL [12]. Other studies, however, show that frailty is indeed associated with increased mortality rates and higher rates of poor HrQoL after TAVI [13,14,15,16]. As such, the impact of preoperative frailty on HrQoL after TAVI still remains unclear. Therefore, this systematic review and meta-analysis aimed to investigate and quantify the impact of frailty on HrQoL in patients after TAVI.

## 2. Materials and Methods

**Figure 1:**
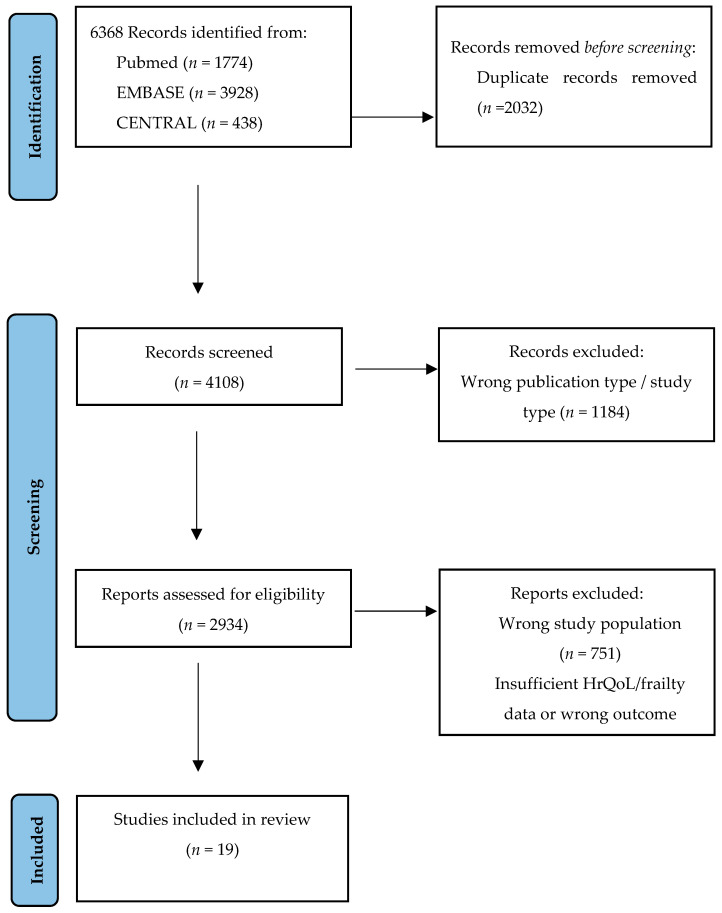
Flowchart of the systematic search and study selection.

The review was performed in line with the preferred reporting items for systematic review and meta-analyses (PRISMA) and by using a predetermined protocol, which was registered with the International Prospective Register of Systematic Reviews (PROSPERO) on the 7 January 2021: CRD42021224894, which can be accessed from https://www.crd.york.ac.uk/prospero/display_record.php?ID=CRD42021224894 [17]. A systematic search of the literature was performed without language restriction. We searched in PUBMED, EMBASE, and the Cochrane Controlled Trials Register (CENTRAL). We searched for clinical trials in adult humans, including randomized controlled trials and observational studies, published from August 2006. The search was conducted until November 2021 and updated in November 2023. The search was performed, using combinations of the following free text terms: TAVI, transcatheter aortic valve implantation, TAVR, transcatheter aortic valve replacement, prognosis, outcome, quality of life, risk prediction, surgical risk and frailty. We considered published full reports of randomized controlled trials and observational human studies that evaluated frailty and HrQoL before and after TAVI. We did not consider data from conference abstracts, letters to the editor, case reports, narrative reviews, animal research, poster presentations or articles from which the full text was not available. We did not perform quality assessments in order to include or exclude the studies. Figure 1 illustrates the flowchart of the systematic search and study selection.

Where multiple studies assessed the same study population, the most complete reporting of outcomes was used, resulting in the exclusion of duplicate publications. We contacted the authors to obtain original data if these were missing or unclear from the publications.

Retrieved reports were screened for inclusion by two authors independently (K.E.H.M.v.d.V. and B.P.A.S.). If consensus could not be reached, a third member of the team was consulted (J.U.S.). The included studies and their characteristics are summarized in Table 1 [3,16,18,19,20,21,22,23,24,25,26,27,28,29,30,31,32,33,34].

In the selected studies, HrQoL was measured by various valid questionnaires. All studies in which patients were defined as frail using a standardized frailty instrument were included. Each author independently scored all eligible reports for methodological validity. The risk of bias was assessed according to the revised Cochrane risk of bias tool for randomized trials (RoB 1.0) and the Newcastle-Ottawa Scale (NOS) for non-randomized studies [35,36]. The NOS score ranges from 0 to 9 points, with a score of ≥7 points considered as high quality and a score of <7 considered as moderate or low quality. Consensus on quality scores and extracted data was reached by discussion. The quality assessment is summarized in Table 2.

Data were analyzed using Review Manager version 5.4.1 (Review Manager. Version 5.4.1. The Cochrane Collaboration, London, UK, September 2020) [37]. Primary analyses were performed by determining means and standard deviations according to the principles of Hozo and colleagues [38,39]. Despite a minimum required number of two studies generally being considered sufficient for meta-analysis, we were expecting significant heterogeneity due to the significant amount of non-randomized studies. Therefore, we determined the requirement of a minimum of three studies for quantitative data synthesis. Due to various types of frailty and HrQoL assessment tools, significant heterogeneity was expected. Therefore, we performed the meta-analysis according to the inverse variance method and the random effects model. Heterogeneity was calculated as a percentage using I^2^ statistics, with >50% indicating significant heterogeneity. Missing *p*-values of continuous variables were calculated by a paired Student’s *t*-test. A *p*-value of 0.05 or less was considered statistically significant.

## 3. Results

### 3.1. Study Characteristics

A total of 951 studies were identified and assessed, leading to a total of 19 studies that were included in this meta-analysis, consisting of 17 cohort studies and 2 randomized controlled trials (RCTs) [3,16,18,19,20,21,22,23,24,25,26,27,28,29,30,31,32,33,34]. The studies consisted of 73,466 patients. In 12 of the 19 studies, patients were considered at high to extreme surgical risk, defined as an estimated risk of mortality or irreversible morbidity within 30 days after SAVR (determined by the Society of Thoracic Surgeons mortality risk model and a multidisciplinary heart team) of, respectively, ≥15% and ≥50%. On the other hand, 7 out of the 19 studies included patients at intermediate surgical risk, defined as an estimated risk of mortality or irreversible morbidity within 30 days after SAVR of 4–8%. Based on the NOS, the overall quality of the non-randomized studies was intermediate (median score of 5, range of 4–8). Based on the RoB 1.0, overall quality of the randomized studies was considered good with a low risk of bias.

### 3.2. Quality of Life

**Figure 2:**
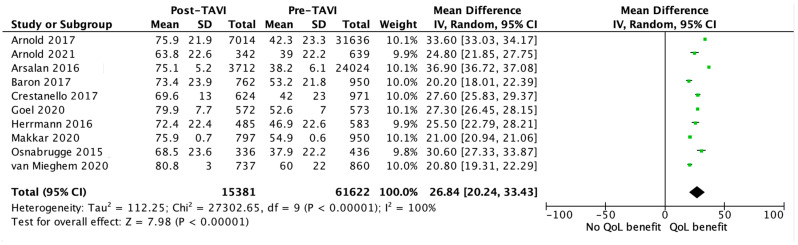
The effect of TAVI on HrQoL after 1 year, measured by the KCCQ [3,18,19,20,21,24,27,31,32,33].

HrQoL was measured by the Kansas City Cardiomyopathy Questionnaire (KCCQ) in 13 of the 19 included studies. Five studies used various forms of the EuroQoL questionnaire (e.g., EQ-5D and EQ-VAS), four studies used the Minnesota living with heart failure questionnaire (MLHFQ), one study used the short form-36 questionnaire (SF-36) and two studies used the SF-12. In most studies using the SF-12 or SF-36, the authors summarized questionnaire data in the physical component scale (PCS) and the mental component scale (MCS). In most studies, the effect of TAVI on HrQoL was measured 1 month and 1 year after the intervention. Quantitative analyses of KCCQ scores in the overall study populations 1 month after TAVI are illustrated in Figure S1 in the Supplementary Materials. This figure shows a mean increase in KCCQ scores of 21.93 (14.05, 29.80) points. This effect persisted during the first year after TAVI, with a mean increase in KCCQ scores after 1 year of 22.75 (22.69, 22.81) points compared to the baseline, as illustrated in Figure 2.

**Figure 3:**
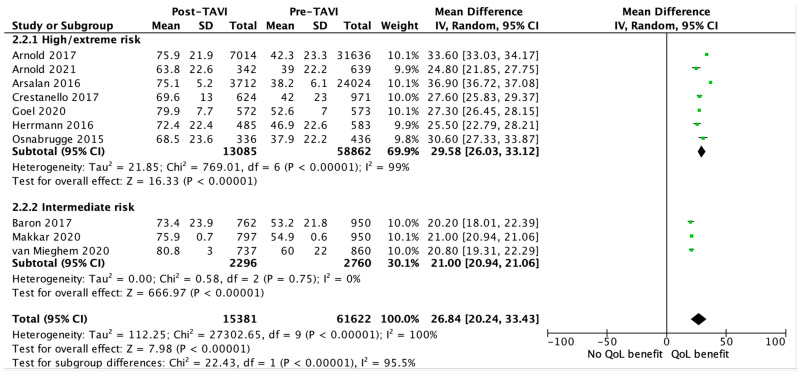
Effect of TAVI on HrQoL according to surgical risk, measured by the KCCQ [3,18,19,20,21,24,27,31,32,33].

These results are consistent with studies that used the MLHFQ to assess HrQoL at 1 month and 1 year after TAVI, as is shown in Supplementary Materials’ Table S1. All the remaining studies, using SF-12, SF-36, or EQ-5D/EQ-VAS, also showed statistically significant improvements after TAVI, as is shown in the Supplementary Materials’ Table S2. The only exception was the study of Frantzen and colleagues, which showed no significant difference in the SF-12 MCS 6 months after TAVI [26]. In the sub-analysis, shown in Figure 3, comparing HrQoL benefits of patients with high to extreme surgical risk with patients with an intermediate surgical risk, we found that both benefitted from TAVI, although the HrQoL benefit was significantly larger in patients with high to extreme surgical risk versus intermediate surgical risk, with a mean increase in KCCQ scores of 29.58 (26.03, 33.12) versus 21.00 (20.94, 21.06) (*p* < 0.00001), respectively.

### 3.3. Frailty

**Figure 4:**
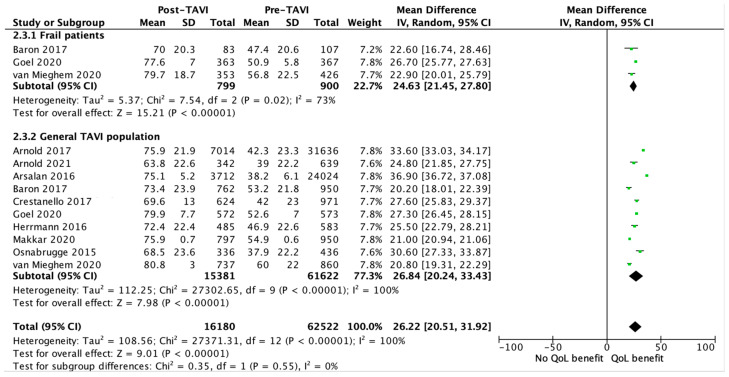
Effect of TAVI on HrQoL in frail patients, measured by the KCCQ [3,18,19,20,21,24,27,31,32,33].

Frailty was measured using a variety of diagnostic tools, in which 14 of the 19 studies used a combination of tools. The 5-meter walk test (5-MWT) was the most frequently used tool for the assessment of frailty (used in 12 of the 19 studies) [3,16,18,19,20,21,25,27,30,32,33,34], followed by grip strength (7 studies) [16,24,25,27,30,33,34], albumin (7 studies) [3,19,21,24,30,33,34], the Katz index (7 studies) [16,19,25,27,29,30,32] and 6-minute walking distance (6-MWD) (5 studies) [28,31,32,33,34]. Other, less frequently used tools for frailty included body mass index (BMI), mini-mental state examination (MMSE), frequent falls, anemia, wheelchair-bound, and weight loss. We received original data from the PARTNER 2 RCT, the SURTAVI trial, and the REPRISE 3 trial, allowing us to perform a meta-analysis of HrQoL data in 900 solely frail patients [21,27,32]. All three studies used the 5-MWT as an indicator of frailty and the KCCQ score for HrQoL assessment. Figure 4 shows consistent and statistically significant HrQoL benefits in frail patients, with a mean KCCQ score increase of 24.6 [21.45, 27.80] (*p* = 0.02). This HrQoL benefit is comparable to the general TAVI population (*p* = 0.55).

Additional data obtained by contacting the authors of three non-randomized studies allowed us to perform a quantitative analysis assessing HrQoL in 270 frail patients undergoing TAVI [16,23,24]. These studies used different HrQoL questionnaires and frailty assessment tools, as is shown in the Supplementary Materials’ Table S3. Only two out of three studies showed benefits to HrQoL that were identified as statistically significant and clinically meaningful [16,23]. Improvement was detected for the physical component domains of SF-36 and SF-12 questionnaires, whereas, 6–12 months after TAVI, no improvement was observed for the mental component domains of the SF-36 and SF-12. The study of Boureau et al. even showed a statistically significant and clinically meaningful decrease in the SF-36 MCS score after 6 months in patients ≥75 years old after TAVI [23].

## 4. Discussion

This systematic review and meta-analysis showed significant HrQoL benefits, not only of statistical but also clinical relevance, in the general TAVI population. In addition, a sub-analysis among 900 frail patients in three RCTs contributed to this quantitative synthesis. When considering a 5-point increase in a KCCQ score as clinically relevant, a mean increase in KCCQ of 24.6 points in the frail subpopulation of these three RCTs is considered a large to very large clinical improvement [40]. These results are consistent with the findings of prior studies [4,6,41]. The beneficial effects of TAVI on HrQoL were independent from the HrQoL questionnaire used or the follow-up period. In addition, our sub-analysis in patients at high to extreme surgical risk versus intermediate surgical risk also showed clinically significant HrQoL benefits in both groups, although patients considered to have an intermediate surgical risk had significantly less HrQoL benefits from TAVI when compared to patients who were considered to have high to extreme surgical risk. We postulate that this can be explained by the fact that patients with an intermediate surgical risk had higher baseline KCCQ scores, resulting in less room for improvement when compared to patients with high to extreme surgical risk, who experienced much lower KCCQ scores at baseline. Another possible explanation to consider is survivorship bias, as a smaller proportion of patients passed away in the intermediate surgical risk group within one year following TAVI. Meanwhile, in the high to extreme risk category, a substantial fraction (at least 10–15%) did not survive past the first year [2,3]. This means that the remaining patients represented a comparatively healthier subset of this population. In the cohort and non-randomized studies that focused on sub-analysis of frail patients, there was no observed benefit to HrQoL. This contrasts with findings from the sub-analysis of frail patients in the three RCTs. We believe this can be explained by the fact that the three studies that were quantitatively analyzed were all RCTs or a substudy of a RCT, representing a particularly selected TAVI population, in comparison to the cohort and non-randomized studies in the latter qualitative analysis. Secondly, possible differences in the sensitivity and specificity of the various instruments used for both the HrQoL and frailty assessments may also play a role in these contradictive results, since little is known about the validity of these tools specifically for TAVI patients. Concerning the different tools that were used for the assessment of HrQoL, a meta-analysis that compared the MLHFQ with KCCQ primarily supported the use of the MLHFQ, followed by the KCCQ, and also demonstrated a correlation between the KCCQ and SF-36 [42]. However, this meta-analysis was performed in patients with heart failure, and it therefore remains unclear if the results can be extrapolated to the TAVI population. A more recent retrospective analysis of the PARTNER cohort showed that the KCCQ is a reliable and valid tool for the assessment of HrQoL in patients with severe, symptomatic aortic stenosis [43].

Concerning frailty assessment, even less is known in TAVI patients. Despite the fact that the 5-MWT is the most commonly used tool in clinical practice to assess frailty in TAVI patients, the use of solely this test lacks specificity to discriminate between complex patients, who may or may not experience benefits from TAVI [44,45]. These studies suggest a preference for using multi-domain scales for frailty assessments, of which the clinical frailty scale (CFS), Edmonton Frail Scale (EFS), and frailty phenotype (FP) are generally accepted for frailty assessments of surgical patients. The FRAILTY-AVR study compared several frailty scales, including Fried, Fried+, Rockwood, Short Physical Performance Battery, Bern, Columbia, and the Essential Frailty Toolset (EFT), in patients undergoing TAVI or SAVR [9]. The EFT, a brief four-item scale, encompassing lower-extremity weakness, cognitive impairment, anemia, and hypoalbuminemia, outperformed the other frailty scales in this study. These recommendations are similar with those of a recently published consensus document for the assessment of frailty in general cardiology patients [46].

This meta-analysis has several limitations. The first limitation of this study is the significant heterogeneity between studies (I^2^ varied from 74% to 100%). This could be explained by the application of a variety of tools for the assessment of both HrQoL and frailty, the different durations of follow-up, the majority of the studies being non-randomized, and the broad standard deviations that were identified in the individual studies, indicating the subjective nature of an outcome measurement such as quality of life. Despite the heterogeneity, we observed an overall benefit to the HrQoL of TAVI patients in the randomized studies that was independent from the assessment tool used. Nevertheless, it should be noted that the majority of studies included in this review used the KCCQ, indicating that the observed improvements in HrQoL are probably primarily focused on disease-specific aspects rather than generic HrQoL as assessed by the SF-36 and SF-12 questionnaires. The KCCQ evaluates various dimensions of HrQoL specific to heart failure, encompassing symptoms, physical limitations, social limitations, and overall quality of life perception. Conversely, generic QoL questionnaires provide a broader assessment of the overall health-related quality of life across multiple domains, extending beyond heart failure symptoms and its consequences. In addition, there is a significant selection bias due to high mortality rates in frail and high to extreme risk patients after TAVI versus in the non-frail or intermediate risk patients, which results in a selected population of relatively healthy frail patients who report HrQoL benefits.

Another potential limitation of this meta-analysis is the dichotomization of frailty in the included studies, where frailty is categorized as either frail or non-frail. This approach simplifies the concept of frailty and may fail to capture the diverse range of frailty among patients. Moreover, if different cut-off points were used to dichotomize frailty in different studies, these could introduce bias and potentially impact the findings of the meta-analysis. The majority of the study results subsequently showed positive results, suggesting a possible publication bias. However, the majority of the studies are cohort studies, with an increased risk of confounding factors compared to RCTs. Therefore, we decided not to perform a specific funnel analysis.

Mortality was not considered in our analysis since this was not the aim of this meta-analysis. However, to translate the results of future studies to clinical practice, it is essential to also consider mortality risk in the process of clinical decision-making since previous studies have shown an increased mortality in frail patients [10,30,47]. In addition to the findings of a previous meta-analysis and other clinical studies in patients with Alzheimer’s disease, related dementias, or cognitive dysfunction, the findings of this meta-analysis emphasize the importance of frailty assessment, since frailty on its own should not be considered as a reason for exclusion from TAVI [48,49,50,51]. Future studies should aim to use randomized trials and strengthen homogeneity by uniformly using the preferable combined frailty tools mentioned above, instead of solely using a 5-MWT or 6-MWD, for example. Further research should focus on recognizing the individual patients at risk for unfavorable outcomes to improve patient selection and optimization before TAVI.

## 5. Conclusions

In conclusion, this systematic review and meta-analysis shows that TAVI improves HrQoL more in high to extreme risk patients compared to intermediate risk patients. Due to the large heterogeneity between studies and conflicting outcomes in RCT versus non-RCT studies, a firm conclusion concerning the impact of preoperative frailty on postoperative HrQoL in TAVI patients cannot be stated.

## Figures and Tables

**Table 1:** Patient characteristics of the populations in the included studies.

First Author, Year	Country	Design	Population	N	Intervention	Surgical Risk	Frailty Assessment	HrQoL Assessment	Follow-Up	Associations
Arnold, 2017 (STS/ACC TVT registry) [18]	USA	Observational, multicenter cohort study	Severe AoS	31,636 and 7014	TAVI	High to extreme surgical risk	5-MWT	KCCQ	30 days and 1 year	Slower gait speed was associated with worse QoL after 1 year.
Arnold, 2021 (CoreValve US pivotal extreme risk trial) [19]	USA	Prospective, multicenter cohort study	Severe, symptomatic AoS (NYHA II-IV) with AVA ≤ 0.8 cm^2^ or AVA index ≤ 0.5 cm^2^/m^2^ with a mean AV gradient of >40 mmHg or AV velocity of >4 m/s	639	TAVI	Extreme surgical risk	Anemia, hypo-albumin, 5-MWT, wheelchair-bound, KIADLS	KCCQ, SF-12	5 years	QoL is improved, even 5 years after TAVI (based on KCCQ).
Arsalan, 2016 (STS/ACC TVT registry) [20]	USA	Retrospective, multicenter cohort study	Severe AoS	24,025	TAVI	High to extreme surgical risk	5-MWT	KCCQ	1 year	TAVI improved QoL to the same degree in nonagenarians as in ‘’younger’’ patients.
Baron, 2017 (PARTNER 2) [21]	USA	RCT	Severe, symptomatic AoS (AVA ≤ 0.8 cm^2^ or AVA index ≤ 0.5 cm^2^/m^2^ with a mean AV gradient of >40 mmHg or AV velocity of >4 m/s)	2032	TAVI vs SAVR	Intermediate surgical risk	5-MWT, albumin	KCCQ, SF-36, EQ-5D	2 years	TF-TAVI resulted in more QoL improvements than TA-TAVI or SAVR at 1 month, but at 1 or 2 years, differences were not statistically significant.
Bäz, 2020 [22]	Germany	Prospective, single center cohort study	Severe, symptomatic AoS	140	TAVI	Intermediate surgical risk	CFS	HADS, EQ-5D, EQ-VAS	1 year	Preoperative frailty was associated with anxiety, depression, and reduced QoL at 6 weeks and 6 months after TAVI.
Boureau, 2017 [23]	France	Prospective, multicenter cohort study	Age 75 years with symptomatic AoS with AVA of <1.0 cm^2^ or a mean gradient of ≥40 mmHg and the presence of a geriatric assessment	150	TAVI	Intermediate and high surgical risk	CIRS-G, BMI, FAB, TUG, IADL, MMSE, mini-GDS	SF-36	6 months	No significant geriatric predictors were associated with physical QoL decline.
Crestanello, 2017 (CoreValve US pivotal high and extreme risk trials) [24]	USA	Retrospective multicenter cohort study	TAVR patients with chronic lung disease (FEV_1_ < 75% and/or on chronic inhaled/oral bronchodilator therapy)	571	TAVI	High and extreme surgical risk	CCI, anemia, albumin, BMI < 21 kg/m^2^, 5-MWT, fall in past 6 months, weight loss, grip strength, assisted living	KCCQ	3 years	Unplanned weight loss, two ADL deficits, wheelchair-bound, assisted living, and fall in the past 6 months were predictors of no health benefit in patients with moderate/severe CLD.
Dziewierz, 2018 [25]	Poland	Retrospective, single center cohort study	Severe, symptomatic AoS (NYHA II-IV) with AVA £ 0.8 cm^2^	148	TAVI	High and extreme surgical risk	5-MWT, EMS, CSHA, KIADLS, grip strength, seniors at risk scale	EQ-5D-3L	1 year	TAVR led to an improvement in QoL for non-COPD and COPD patients, and COPD patients were frailer and had more pronounced QoL improvements.
Frantzen, 2021 (CARDELIR) [26]	Norway	Prospective, single center cohort study	Age of 80 years with severe, symptomatic AoS (AVA index of <0.6 cm^2^/m^2^, a mean AV gradient of ≥40 mmHg and peak AV velocity of ≥4 m/s)	143	TAVI or SAVR	High surgical risk	SOF index, MMSE, BMI	SF-12, MLHFQ	6 months	Frail patients had a significantly lower QoL both before and after TAVI/SAVR; however, there was a significant reduction of frailty after TAVI/SAVR.
Goel, 2020 (REPRISE 3) [27]	USA	Substudy of a TAVR cohort	Severe, symptomatic AoS (AVA £ 1.0 cm^2^ or AVA index of ≤0.6 cm^2^/m^2^ and a mean AV pressure gradient of 40 mmHg or AV jet velocity of 4 m/s)	576	TAVI	High and extreme surgical risk	5-MWT, frailty, grip strength, KIADLS, mini-COG	KCCQ	1 year	Faster gait speed was associated with better HrQoL 1 year after TAVI.
Gotzmann, 2011 [28]	Germany	Prospective, single center cohort study	Age of ≥75 years old with AVA < 1 and a EuroSCORE ≥ 15% or >60 years old with additional specified risk factors	51	TAVI	High and extreme surgical risk	6-MWD	MLHFQ	1 year	TAVI improved HrQoL and reduced frailty.
Gouda, 2020 [29]	Canada	Prospective, single center cohort study	Severe, symptomatic AoS	171	TAVI	High to extreme surgical risk	10-MWT, KIADLS	MLHFQ	1 year	Despite improvements in HrQoL after TAVI, no improvements in frailty were observed in patients at 1 year.
Goudzwaard, 2020 [16]	The Netherlands	Prospective, single center cohort study	Age of ≥70 years with severe, symptomatic AoS	239	TAVI	Intermediate and high to extreme surgical risk	Erasmus frailty score, CIRS-G, MMSE, grip strength, MUST, KIADLS, Lawton and Brody index, TUG, 5-MWT	EQ-5D-5L	1 year	Frailty was associated with the deterioration of HrQoL one year after TAVI.
Green, 2015 (PARTNER) [30]	USA	Retrospective, multicenter cohort study	Severe, symptomatic AoS, AVA < 0.8 cm^2^ and a mean AV pressure gradient of 40 mmHg or AV jet velocity of 4 m/s	244	TAVI	High to extreme surgical risk	Albumin, KIADLS, grip strength, 5-MWT	KCCQ	1 year	Frailty was associated with decreased HrQoL 1 year after TAVI.
Herrmann, 2016 (PARTNER 2 SAPIEN 3) [31]	USA	Prospective, multicenter cohort study	Severe, symptomatic AoS, AVA ≤ 0.8 cm^2^ and a mean AV pressure gradient of 40 mmHg or AV jet velocity of 4 m/s	583	TAVI	High to extreme surgical risk	6-MWD	KCCQ	1 year	TAVI led to an improvement in HrQoL and a reduction in frailty parameters.
Makkar, 2020 (PARTNER 2) [3]	USA, Canada	RCT	Severe, symptomatic AoS, AVA ≤ 0.8 cm^2^ and a mean AV pressure gradient of 40 mmHg or AV jet velocity of 4 m/s	2032	TAVI or SAVR	Intermediate surgical risk	5-MWT, albumin	KCCQ	5 years	Both TAVI and SAVR led to an improvement in HrQoL.
Van Mieghem, 2020 (SURTAVI) [32]	The Netherlands	RCT	Severe, symptomatic AoS with AVA ≤ 1 cm^2^ or AVA index < 0.6 cm^2^/m^2^ and a mean AoV PG of >40 mmHg or maximum AV velocity of >4 m/s	1660	TAVI or SAVR	Intermediate surgical risk	6-MWD, 5-MWT, falls in past 6 months, BMI < 21, KIADLS, MMSE	KCCQ	2 years	Both TAVI and SAVR led to an improvement in HrQoL, but HrQoL improved faster with TAVI compared to SAVR. Frailty was significantly decreased with TAVI and remained significantly better with TAVI over SAVR at 2 years.
Osnabrugge, 2015 (CoreValve US extreme risk trial) [33]	USA	Prospective, multicenter cohort study	Severe, symptomatic AoS with a 30-day risk of mortality/irreversible morbidity ≥50%	471	TAVI	Extreme surgical risk	Albumin, wheelchair-bound, 6-MWD, 5-MWT, grip strength, CCI	KCCQ, SF-12, EQ-5D	1 year	Preoperative wheelchair-bound patients and patients with baseline low HrQoL had a higher risk of poor outcomes (decrease/no increase in HrQoL).
Søndergaard, 2018 (PORTICO-I) [34]	International (61 sites in Europe, Australia, and Canada)	Prospective, multicenter cohort study	Severe, symptomatic AoS	941	TAVI	Intermediate surgical risk	5-MWT, 6-MWD, grip strength, albumin, MMSE, unintentional weight loss	EuroQoL visual analogue scale	1 year	Functional class, exercise capacity, and HrQoL significantly improved from baseline to 1 year after TAVI.

5-MWT = 5-meter walk test; 6-MWD = 6-minute walking distance; AoS = aortic valve stenosis; AoV = aortic valve; AVA = aortic valve area; BMI = body mass index; CCI = Charlson comorbidity index; CFS = clinical frailty scale; CIRS-G = Cumulative Illness Rating Scale for Geriatrics; COPD = chronic obstructive pulmonary disease; CSHA = Canadian Study of Health and Aging scale; EMS = Elderly Mobility Scale; EQ-5D = Euro Quality of Life 5 dimensions; FAB = frontal assessment battery; HADS = Hospital Anxiety and Depression Scale; IADL = Lawton instrumental activities of daily living scale; KCCQ = Kansas City Cardiomyopathy Questionnaire; KIADLS = Katz index of independence in activities of daily living score; mini-COG = mini-cognitive assessment for dementia score; mini-GDS = mini-Geriatric Depression Scale; MLHFQ = Minnesota living with heart failure questionnaire; MMSE = mini-mental status examination; RCT = randomized controlled trial; SAVR = surgical aortic valve replacement; SF-12 = short form-12; SOF index = study of osteoporotic fracture index; STS = Society for Thoracic Surgeons risk score; TAVI = transcatheter aortic valve implantation; TUG = Timed Up and Go test; USA = United States of America.

**Table 2:** Quality assessment of the individual studies.

**Randomized Controlled Trials ^a^**									
**Publication**	**Selection bias** Random sequence generation	**Selection bias** Allocation concealment	**Reporting bias**	**Other bias**	**Performance bias**	**Detection bias**		**Attrition bias**	**Total score**
Baron, 2017 [21]	Unclear	Unclear	Low risk of bias	Low risk of bias	Unclear	Low risk of bias		Low risk of bias	Low risk of bias
Makkar, 2020 [3]	Unclear	Unclear	Low risk of bias	Low risk of bias	Low risk of bias	Low risk of bias		Low risk of bias	Low risk of bias
**Observational Studies (Cohort Studies) ^b^**									
	**Selection**				**Comparability**	**Outcome**			**Total score**
Publication	Representativeness of exposed cohort	Selection of non-exposed cohort	Ascertainment of exposure	Demonstration: outcome was not present at start	Controls and adjusted	Assessment of outcome	Was follow-up long enough	Adequacy of follow-up of cohorts	
Arnold, 2017 [18]	1	0	1	1	0	0	1	1	5
Arnold, 2021 [19]	1	0	1	1	0	0	1	1	5
Arsalan, 2016 [20]	1	1	1	1	2	0	1	0	7
Bäz, 2020 [22]	1	0	1	1	0	0	1	1	5
Boureau, 2017 [23]	1	0	1	1	0	0	1	1	5
Crestanello, 2017 [24]	1	0	1	1	0	0	1	1	5
Dziewierz, 2018 [25]	1	1	1	1	2	0	1	1	8
Frantzen, 2021 [26]	1	1	1	1	2	1	1	0	8
Goel, 2020 [27]	1	0	1	1	0	0	1	1	5
Gotzmann, 2011 [28]	1	0	1	1	0	0	1	0	4
Gouda, 2020 [29]	1	0	1	1	0	0	1	0	4
Goudzwaard, 2020 [16]	1	1	1	1	1	0	1	1	7
Green, 2015 [30]	1	1	1	1	0	1	1	0	6
Herrmann, 2016 [31]	1	0	1	1	0	0	1	0	4
Osnabrugge, 2015 [33]	1	0	1	1	0	0	1	1	5
Søndergaard, 2018 [34]	1	0	1	1	0	0	1	1	5
Van Mieghem, 2020 [32]	1	0	1	1	0	1	1	0	5

^a^ Cochrane risk of bias (RoB 1.0). ^b^ Quality assessment using the Newcastle-Ottawa Scale (NOS) (ref). Total score = 9; studies were rated as good with a score of ≥7, moderate with a score of 5–6, and poor with a score of ≤4.

## Data Availability

Data are contained within the article and Supplementary Materials.

## Supplementary Materials

The following supporting information can be downloaded at: https://www.mdpi.com/article/10.3390/jcdd11100333/s1, Table S1: Health-related quality of life measured by MLHFQ after TAVI; Table S2: Health-related quality of life measured by remaining tools after various periods of follow-up; Table S3: Quality of life of frail patients measured by remaining tools after TAVI; Figure S1: Health-related quality of life measured by KCCQ 1 month after TAVI.

## Abbreviations

5-MWT = 5-meter walk test; 6-MWD = 6-minute walking distance; AS = aortic stenosis; HrQoL = health-related quality of life; KCCQ = Kansas City Cardiomyopathy Questionnaire; MLHFQ = Minnesota living with heart failure questionnaire; RCTs = randomized controlled trials; SAVR = surgical aortic valve replacement; SF-12 = short form-12 questionnaire; SF-36 = short form-36 questionnaire; TAVI = transcatheter aortic valve implantation.
