# Supplementary material for: The Impact of Frailty and Surgical Risk on Health-Related Quality of Life After TAVI
Source: J Cardiovasc Dev Dis. 2024 Oct 18;11(10):333. doi: 10.3390/jcdd11100333 (PMC11508619; doi:10.3390/jcdd11100333)

# The impact of frailty and surgical risk on health-related quality of life after TAVI

**A systematic review and meta-analysis**

## Supplementary Materials

**Supplementary Materials**

*Table S1. Health-related quality of life, measured by MLHFQ after TAVI.*

*Table S1a indicates the effect of TAVI on HrQoL, measured by MLHFQ after 30 days.*

| Study | Pre-TAVI | | | Post-TAVI | | | |
|---|---|---|---|---|---|---|---|
| | N | Mean | SD | N | Mean | SD | *p* |
| Gotzmann 2011 [28] | 51 | 39,6 | 19 | 51 | 26,1 | 18 | *< 0.001* |
| Gouda 2020 [29] | 82 | 32,2 | 15,6 | 82 | 13,2 | 11,5 | *< 0.001* |

*Table S1b indicates the effect of TAVI on HrQoL, measured by MLHFQ, after 1 year.*

| Study | Pre-TAVI | | | Post-TAVI | | | |
|---|---|---|---|---|---|---|---|
| | N | Mean | SD | N | Mean | SD | *p* |
| Gotzmann 2011 [28] | 51 | 39,6 | 19 | 51 | 26,1 | 18 | *< 0.001* |
| Gouda 2020 [29] | 41 | 28,8 | 15 | 82 | 17,3 | 16,9 | *0.001* |

*Table S2. Health-related quality of life, measured by remaining tools, after various periods of follow-up*

| Study | HrQoL questionnaire | Follow-up | Pre-TAVI | | | Post-TAVI | | | |
|---|---|---|---|---|---|---|---|---|---|
| | | | N | Mean | SD | N | Mean | SD | |
| Arnold 2021 [19] | SF-12 PCS | 1 year | 639 | 28,4 | 8,2 | 318 | 33,2 | 9,6 | |
| | SF-12 MCS | 1 year | 639 | 46,2 | 12,2 | 318 | 48,6 | 11,4 | |
| Baron 2017 [21] | SF-36 PCS | 1 year | 950 | 36,0 | 8,9 | 746 | 40,3 | 2,3 | [illegible] |
| | SF-36 MCS | 1 year | 950 | 48,7 | 11,3 | 746 | 51,7 | 3,2 | [illegible] |
| | EQ-5D | 1 year | 950 | 0,75 | 0,2 | 754 | 0,79 | 0,1 | [illegible] |
| Boureau 2017 [23] | SF-36 PCS | 6 months | 150 | 33,6 | 8,4 | 119 | 36,4 | 9,96 | |
| | SF-36 MCS | 6 months | 150 | 55,6 | 8,1 | 119 | 48,2 | 9,44 | |
| Dziewierz 2018 [25] | EQ-VAS | 1 year | 148 | 24,1 | 17,6 | 116 | 68,8 | 11,1 | [illegible] |
| Frantzen 2021 [26] | SF-12 PCS | 6 months | 124 | 34,0 | 10,5 | 96 | 40,1 | 10,5 | [illegible] |
| | SF-12 MCS | 6 months | 124 | 48,7 | 11 | 96 | 51,4 | 9,3 | |
| Goudzwaard 2020 [16] | EQ-5D | 1 year | 235 | 0,66 | 0,3 | 196 | 0,73 | 0,24 | [illegible] |
| Osnabrugge 2015 [33] | SF-12 PCS | 1 year | 436 | 28,5 | 8,3 | 366 | 34,1 | 10,6 | |
| | SF-12 MCS | 1 year | 436 | 45,8 | 12,3 | 366 | 51,7 | 11,8 | |
| | EQ-5D | 1 year | 436 | 0,65 | 0,24 | 366 | 0,73 | 0,2 | |
| Sondergaard 2018 [34] | EQ-VAS | 1 year | 620 | 62,2 | 18,8 | 620 | 69 | 17,9 | [illegible] |

*Table S3. Quality of life of frail patients, measured by remaining tools, after TAVI*

| **Study** | **Frailty measurement** | **Follow-up** | **HrQoL questionnaire** | **Pre-TAVI** | | | **Post-TAVI** | |
|---|---|---|---|---|---|---|---|---|
| | | | | N | Mean | SD | N | Mean |
| Boureau 2017 [23] | TUG | 6 months | SF-36 PCS | 119 | 33,7 | 8,5 | 119 | 36,4 |
| | TUG | 6 months | SF-36 MCS | 119 | 55,6 | 8,2 | 119 | 48,2 |
| Goudzwaard 2020 [16] | SOF-index | 1 year | SF-12 PCS | 81 | 32,2 | 9,6 | 53 | 35,8 |
| | SOF-index | 1 year | SF-12 MCS | 81 | 47,1 | 11,8 | 53 | 48,5 |
| Sondergaard 2018 [34] | Erasmus frailty score | 1 year | EQ-5D | 70 | 0,55 | 0,26 | 51 | 0,44 |
| | Erasmus frailty score | 1 year | EQ-VAS | 70 | 58,4 | 16,8 | 51 | 63,4 |

*Figure S1. Health-related quality of life, measured by KCCQ, 1 month after TAVI.*

| **Study or Subgroup** | **post-TAVI Mean** | **SD** | **Total** | **pre-TAVI Mean** | **SD** | **Total** | **Weight** | **Mean Difference IV, Random, 95% CI** |
|---|---|---|---|---|---|---|---|---|
| Arnold 2017 [18] | 69.9 | 23.7 | 31636 | 42.3 | 23.7 | 31636 | 14.4% | 27.60 [27.23, 27.97] |
| Arnold 2021 [19] | 58.8 | 22.3 | 332 | 39 | 22.2 | 639 | 14.1% | 19.80 [16.85, 22.75] |
| Arsalan 2016 [20] | 67.8 | 5.1 | 10257 | 38.2 | 6.1 | 24025 | 14.5% | 29.60 [29.47, 29.73] |
| Baron 2017 [21] | 68.2 | 25.1 | 874 | 53.2 | 21.8 | 950 | 14.3% | 15.00 [12.83, 17.17] |
| Makkar 2020 [3] | 70.3 | 0.6 | 913 | 54.9 | 0.6 | 950 | 14.5% | 15.40 [15.35, 15.45] |
| Osnabrugge 2015 [33] | 62 | 25.8 | 253 | 37.9 | 22.2 | 436 | 13.9% | 24.10 [20.30, 27.90] |
| van Mieghem 2020 [32] | 78.3 | 5.8 | 819 | 60 | 22 | 860 | 14.4% | 18.30 [16.78, 19.82] |
| **Total (95% CI)** | | | **45084** | | | **59496** | **100.0%** | **21.40 [14.14, 28.67]** |

Heterogeneity: $Tau^2 = 95.16$; $Chi^2 = 44259.12$, df = 6 ($P < 0.00001$); $I^2 = 100\%$
Test for overall effect: $Z = 5.77$ ($P < 0.00001$)

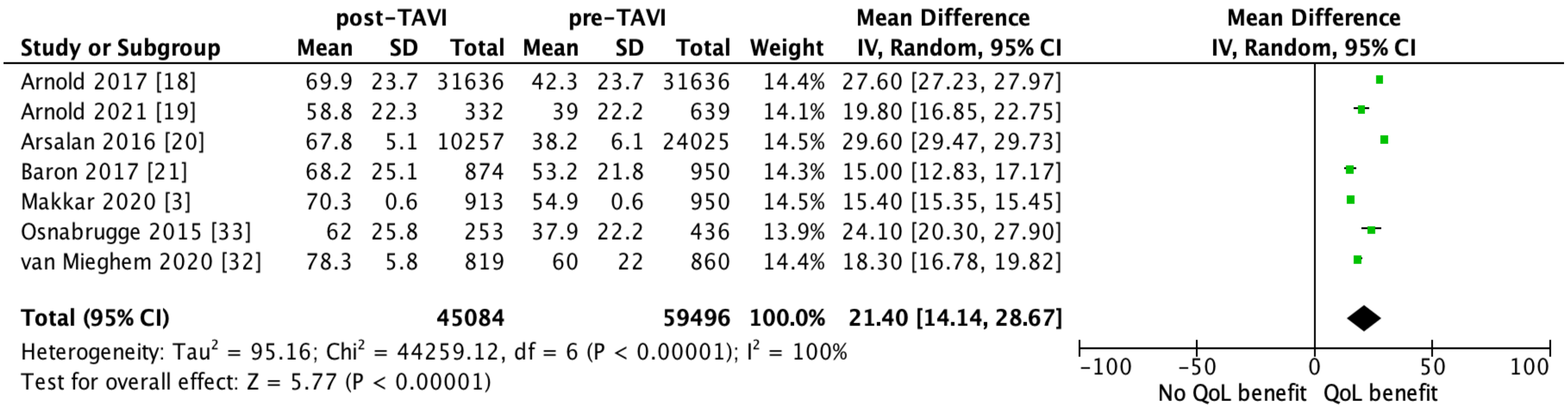

Supplement: Supplementary file 1 [file jcdd-11-00333-s001.zip › jcdd-3152622-supplementary-final.pdf]
